# Worldwide study on field trials of biotechnological crops: new promises but old policy hurdles

**DOI:** 10.3389/fpls.2024.1452767

**Published:** 2024-11-04

**Authors:** Agnès Ricroch, Louie-David Desachy, Mateo Penfornis, Melekşen Akin, Ankica Kondić-Špika, Marcel Kuntz, Dragana Miladinović

**Affiliations:** ^1^ Laboratoire Institut Droit, Espaces et Technologies (IDEST), Faculté Jean Monnet, Université Paris-Saclay, Sceaux, France; ^2^ AgroParisTech, Université Paris-Saclay, Palaiseau, France; ^3^ Department of Horticulture, Agricultural Faculty, Iğdır University, Iğdır, Türkiye; ^4^ Laboratory for Biotechnology, Institute of Field and Vegetable Crops, National Institute of Republic of Serbia, Novi Sad, Serbia; ^5^ Laboratoire de Physiologie Cellulaire et Végétale, Université Grenoble Alpes, Centre national de la recherche scientifique (CNRS), Commissariat à l'énergie atomique et aux énergies alternatives (CEA), Institut national de recherche pour l'agriculture, l'alimentation et l'environnement (INRAE), Grenoble, France

**Keywords:** transgenesis, CRISPR, genome editing, plant breeding, field trials, biotechnology regulatory policy

## Abstract

Field trials (FTs) are a necessary step towards future commercialization of biotech crops and products thereof, whether for research and development or cultivation approval. A total of 187 FTs in 30 countries have been compiled for 2022 and 2023 using a survey and intergovernmental databases. FTs have been classified according to methods, crops and traits. Compiled FTs are mostly conducted by the public sector on eight plant species with improved stress resistance, industrial application, yield, and quality. Regarding genome editing (GenEd), 23 FTs (12% of total) are carried out in 6 countries, on 10 crops. Regulations were examined in 141 countries to discuss why in some countries FTs are not performed, although basic biotech research is carried out. The EU particularly is compared to the rest of the world. Regarding the new proposal in the EU for GenEd product classification, it was found that all recent FTs of such products fall in the category that the EU would consider as ‘equivalent to conventional plants’ (NGT-1). We also studied current cultivation approvals to highlight differences with crops tested in the field and those may be approved in the future.

## 1 Introduction

Field trials (FTs) often have a crucial importance for both research in plant breeding and for compliance with relevant risk assessment policies. This is illustrated by recent articles dealing with rice (Andrew-Peter-Leon et al., 2021; Hu et al., 2019; Zhang et al., 2021), tomato (Chandrasekaran et al., 2021), trees (Chanoca et al., 2019; Lu et al., 2017), banana (Dale et al., 2017), barley (Cheng et al., 2023), citrus (Huang et al., 2018), poplar (Jang et al., 2021), soybean (Kambhampati et al., 2017), wheat (Raffan et al., 2023), maize (Gao et al., 2020; Simmons et al., 2021), camelina (Han et al., 2022), potato (Hofvander et al., 2022), and cotton (Perumalla et al., 2018), that stressed the importance of FTs for research. However, there are heterogeneous regulatory policies in the world with respect to the acceptance of biotech techniques for crop improvement including FT policies (Metje-Sprink et al., 2020; Schiemann et al., 2020; Sprink and Wilhelm, 2024). Hence, although genome editing (GenEd) using CRISPR-Cas9 has been used in plant breeding since 2014, only 3 crops are commercialized up to now. Independently of the necessity of performing FTs to comply with specific (risk) regulation imposed on biotech crop, deregulated crops also need to be assessed by FTs in some countries, for example in countries (e.g. European countries) which comply to the UPOV system of Proprietary Plant Variety Protection Certificates (COV): it is mandatory to perform FTs under various geographical environments in order to obtain such a certificate which is a prerequisite for marketing.

The points mentioned above show that FTs are important (and often mandatory) for various reasons. Therefore in present study, we compiled the information on the ongoing FTs with biotech crops worldwide, using our own survey encompassing 55 countries which was completed by a survey and by screening available databases. We wanted to determine what countries are pursuing laboratory (confined) research and whether it was followed by FTs, and for which traits. As FTs are a step upstream to the acceptance of an event for commercialization, we completed our study by an analysis of events approved for cultivation between 2021 and 2023. These approvals were considered the result of FTs that occurred upstream and show innovation close to the market release of new plants, traits, and biotech events. A particular attention was paid to GenEd (CRISPR-Cas9 or Talen) compared with transgenesis (Agrobacterium mediated gene transfer and RNAi).

Furthermore, we also examined to what extent regulation influences FTs acceptance, since in the European Union (EU) for example, under Directive 2015/412 Member States (MS) can either restrict or prohibit GMO cultivation on their territory and this Directive was found to have a direct effect on FTs in some MS (Ricroch, 2020). In addition, we performed a bibliometric analysis on recent publications mentioning FTs to provide a holistic overview of the fragmented literature on the topic by delineating research hotspots and hidden network patterns between scientific actors including countries and institutions.

## 2 Methods

### 2.1 Survey

We conducted a survey of over 55 countries in 2022-2023 contacting researchers competent in the domain of biotech plants, either transgenic (Tr) or genome edited (GenEd) (**Supplementary Methods S1** file of **Supplementary File**), which was completed with numerous databases: the governmental site of each country when the data were available, the website of the European Commission (EC), the USDA GAIN (November 2022 for the most recent report) and the FAO survey (2016-2022), OECD and BCH for Cartagena Protocol.

FTs performed for several years are considered once (the year they were initiated). From 2015 to mid-2023 we examined the trends up based on previous data (Ricroch, 2020). Thus, UK is included in this update as it was part of the EU until 31 January 2020. GenEd crops have been tested in the EU since 2017 while they were tested before as in the USA. We considered the date of acknowledgement from the Member State Competent Authority as the beginning of the FT as in the previous study.

For cultivation approvals, we examined all events recorded in the ISAAA GM approval database (which includes biotech events that have been approved for commercialization/planting and importation at https://www.isaaa.org/gmapprovaldatabase/) and in governmental databases (the databases were used by the researcher interviewed as part of the survey).

The term ‘plant species’ is used both for cultivated plants and model plants (*Arabidopsis*).

### 2.2 Bibliometric data extraction and analysis

To extract bibliographic data from the WoS and Scopus databases, we used the following search strings within document title, abstract and author keywords:

((“ field tr*” OR “field test*”) AND (“GM” OR “gen* edit*”)) AND (“gene edit*” OR “genome edit*” OR CRISPR OR ODM OR TALEN OR NBT OR NGT OR SDN1 OR SDN2 OR SND3 OR mutat* OR “allele replacement”) NOT (rodent* OR mosqui* OR embryo OR therapy OR virus OR covid OR consum* OR animal* OR human* OR pig* OR *fish*)

The Boolean operators (AND and OR) and wildcards were utilized to find publications with various combinations of the specified keywords in both singular and plural forms. The search was conducted in May 2023, and the syntax resulted in 35 documents in WoS and 36 documents in Scopus. The data from both databases were merged, duplicates were detected and removed (which resulted in 41 documents) utilizing Bibliometrix package in R studio. After that the content of each manuscript was read and 8 irrelevant papers with the topic were discarded. The final data consisted of 33 publications on the field.

The bibliographic data in this work was subjected to bibliometric analysis utilizing Bibliometrix package and Biblioshiny interface in R studio (Aria and Cuccurullo, 2017; R Core Team, 2022). Keyword and network analysis were performed.

## 3 Results and analysis

The present compilation of FTs of biotech plants used a cross-reference of multiple sources (see Methods) to ensure data veracity and recentness. FTs performed in the EU, Iceland, Switzerland, and the UK could be analyzed in more details as these regions disseminate the relevant information openly. However, in spite of the examination of all databases, no data could be obtained for some countries, which prompted us to investigate whether these countries conduct laboratory research on biotech plants (**Supplementary Table S1**).

### 3.1 Worldwide landscape of FTs

**Figure 1:**
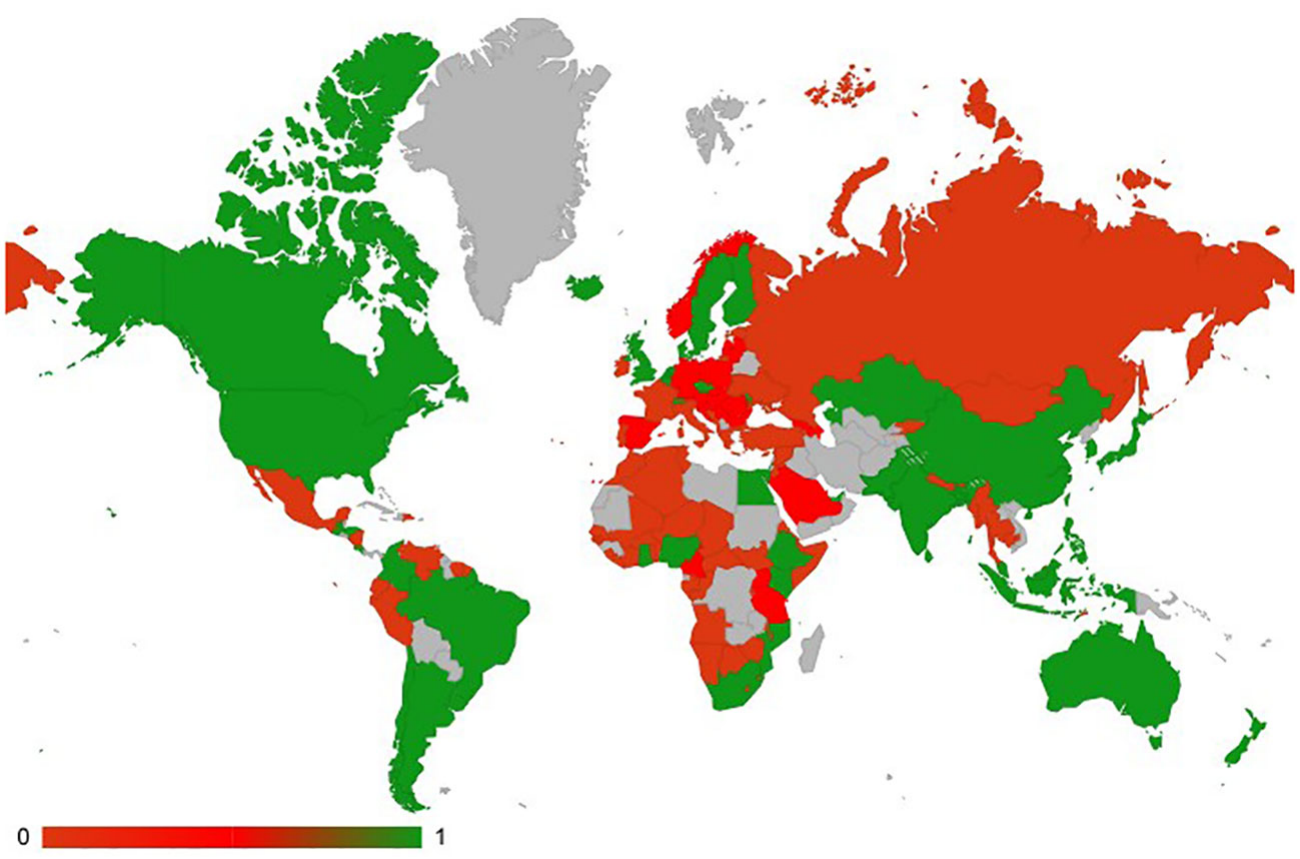
Map of countries with ongoing FTs (green), no ongoing FTs (red), and no public data (grey).

The results of our large-scale study have shown that, out of 193 UN countries, general data on biotech FTs could be obtained for 141 countries (**Supplementary Tables S1, S2** of the **Supplementary File**), including, among the 89 countries, 41 countries with ongoing FTs (green), 48 with no ongoing FTs (red) and 52 with no public data (grey) (see map in **Figure 1**). Further analysis showed that 66 countries authorize FTs on their territory, but only 41 actually have ongoing FTs (**Supplementary Table S2**). The **Supplementary Table S2** listed all the 89 countries including 62 countries outside the EU and the 27 EU member states.

It was also found that in 36 countries FTs with biotech plants are allowed, but additional approval of the authorities is needed, while in only two countries those FTs are allowed without additional approvals (Czech Republic and Egypt). In 11 countries this type of FTs is not allowed or not possible in the local context, with almost half of these countries being members of the EU (Cyprus, Estonia, France, Ireland and Portugal). As is apparent from **Figure 1**, the countries in Asian (including Middle East) and American continents have more favorable attitude to the FTs with biotech crops than the EU and Africa, although in Africa some countries, such as Egypt, Ethiopia, Ghana, Kenya, Mozambique, Nigeria, are currently performing FTs. It should also be noted that in some countries the regulation changed in favor of FT acceptance as in Japan or in Italy in June 2023 (Global Gene Editing Regulation Tracker, 2023). In Spain some requests for FTs in 2023 are pending. In South Africa since 2021 no FTs have been registered in the governmental database (DALRRD, Department of Agriculture, Land Reform and Rural Development). Since our survey also examined whether laboratory research on biotech plants is being performed, we found out that 11 countries carry out laboratory research but do not perform FTs (**Supplementary Tables S1**, **S2**).

In **Supplementary Table S1**, we have listed the countries where laboratory biotechnology research is being carried out, and in **Supplementary Table S2**, the countries where field trials are being conducted. These two tables provide information on the current state of research. Some countries carry out laboratory research but are not authorized to carry out field trials.

**Figure 2:**
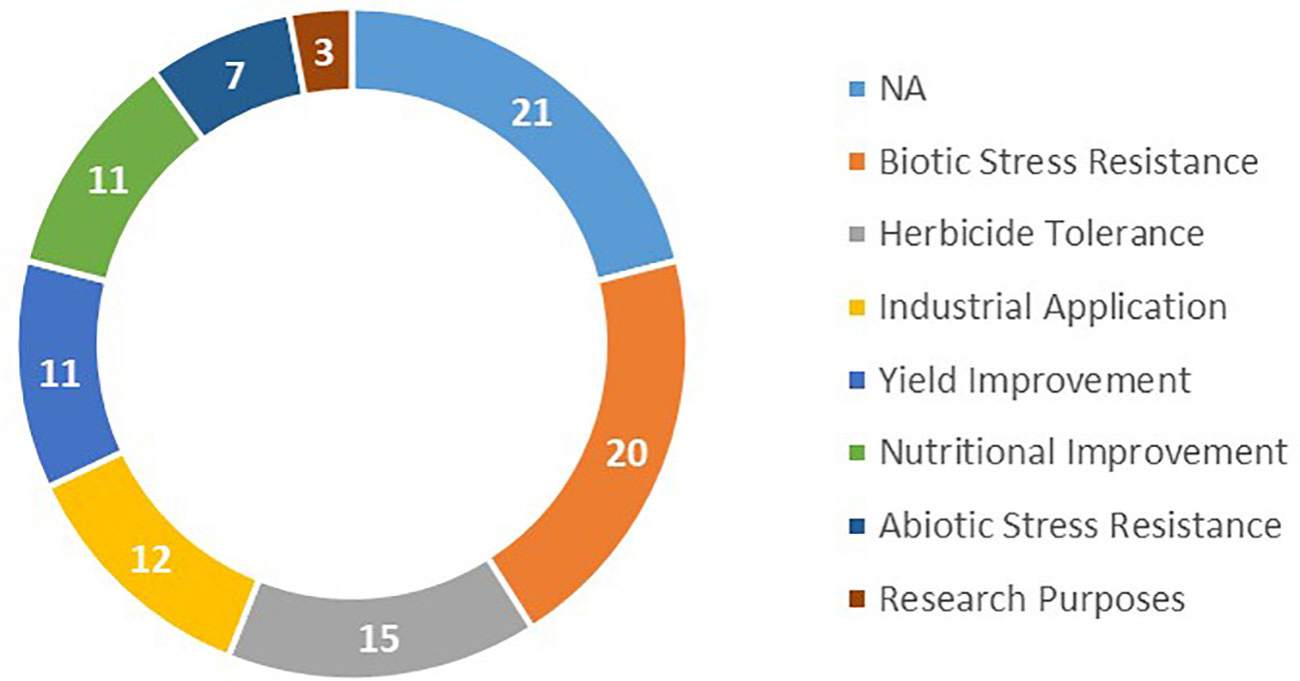
Biotech traits tested in FTs in percentage.

The compiled FTs were further classified according to the traits that have been introduced (**Figure 2**). These traits are related to biotic stress resistance (20%), herbicide tolerance (15%), industrial application (12%), yield (11%), quality improvement (11%), abiotic stress resistance (7%), and fundamental research with model plant (3%).

Data obtained in our work showed that canola and maize are the most field-tested crops for herbicide tolerance (**Supplementary Table S3**). We identified ‘new’ herbicide tolerant crop species tested in FTs, that is the ones not already approved for cultivation (see section 3.3). These ‘new’ species are bent grass, pine trees, and wheat. Regarding countries, most FTs of herbicide tolerant crops are conducted in Asia-Pacific region. However, Canada stands out with 5 different crops tested (camelina, canola, maize, soybean, wheat).

Our study showed that six non-EU countries field-tested abiotic stress resistance and only one EU country (Belgium) performed such FT (**Supplementary Table S4**). ‘New’ crops tested are barley, chickpea, poplar, rice, and turf grass, mostly for drought tolerance. Resistance to biotic stress is by far the most tested trait in current FTs (**Supplementary Table S5**). Those took place in all five continents. Potatoes are largely represented for this trait, with occurrences in 6 different countries and one FT with GenEd potatoes in Sweden.

Quality improvement FTs are mostly related to biofortification in ‘new’ crops (apple, beans, wheat) and oil content also in ‘new’ crops (camelina, Indian mustard, wheat) along with canola (**Supplementary Table S6**). In this category, only one variety of maize is edited with CRISPR-Cas9, for decreasing lignin content. As for industrial applications, ten crops in seven countries in the EU, Asia-Pacific and America were tested (**Supplementary Table S7**).

Yield improvement traits were related to the nutritional efficiency and the global architecture of the plant (**Supplementary Table S8**). ‘New’ crops were tested (barley, camelina, pine trees, wheat) for yield. Barley, maize, wheat represent the majority of the FTs in this category. Canada and Asia-Pacific performed most of such FTs, with only one EU country performing FTs along with the UK. In 2022 one FT with yield improved crop was set in Japan for the first time since 2016. Finally, plant species used in FTs for research purposes were Arabidopsis, aspen, grass, pine, poplar, sorghum, and white clover (**Supplementary Table S9**). Most of these FTs are funded by the public sector.

Regarding more specifically GenEd species tested in FTs, they are 10, namely barley (yield in the UK), camelina (yield in the UK), chili (biotic stress resistance in Indonesia), citrus (biotic stress resistance in Indonesia; quality improvement, biotic stress resistance in the USA), potato (biotic stress resistance, industrial applications in Denmark; herbicide tolerance in Indonesia; biotic stress resistance, industrial applications in Sweden), poplar (yield in Sweden; USA), maize (abiotic stress resistance, quality improvement, yield in Belgium; herbicide tolerance in USA), rice (abiotic and biotic stress resistance, yield in Indonesia), soybean (herbicide tolerance in USA), and wheat (yield in UK) (**Supplementary Table S10**).

### 3.2 The current FT situation in Europe

#### 3.2.1 Crops and traits

As GenEd is becoming a mature and increasingly used technology, researchers are faced with the necessity to evaluate their new crops in field growing conditions. However, knowing that the EU has very strict regulations on GMO cultivation (since 1998 it only authorized a Bt maize and a potato which is no longer marketed), it is interesting to compare the 27 MS of the EU and other countries in Europe with the rest of the world to evaluate the impact of regulation on innovation, as revealed by FTs. It is also interesting to analyze whether MS conduct field trials for proof of concept or for risk assessment at the EU level. Another question is whether some MS are more permissive than others regarding FTs since the ‘opt-out’ Directive (EU) 2015/412.

**Figure 3:**
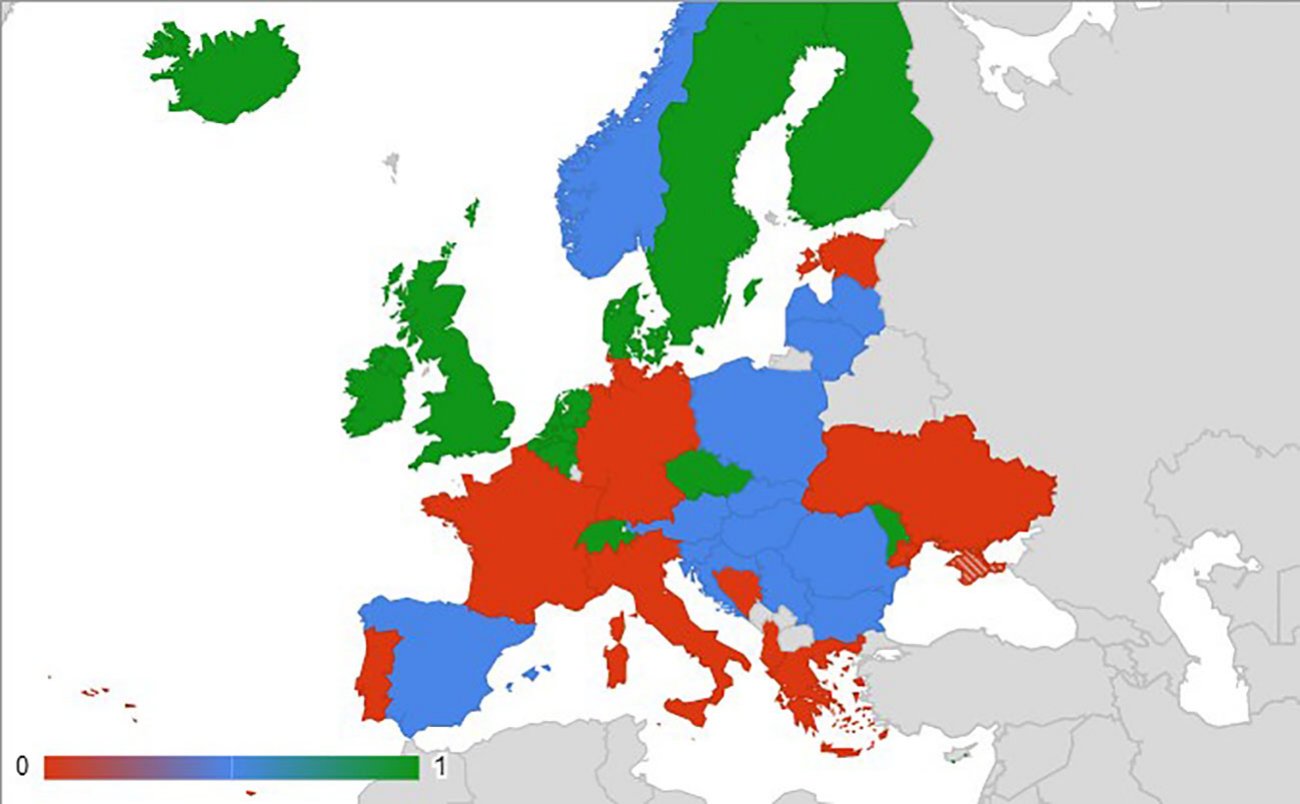
Map of countries in Europe where FTs are ongoing (green), allowed (blue), forbidden or currently non-existing (red). Cyprus, not seen on the map, is pictured in red.

**Figure 4:**
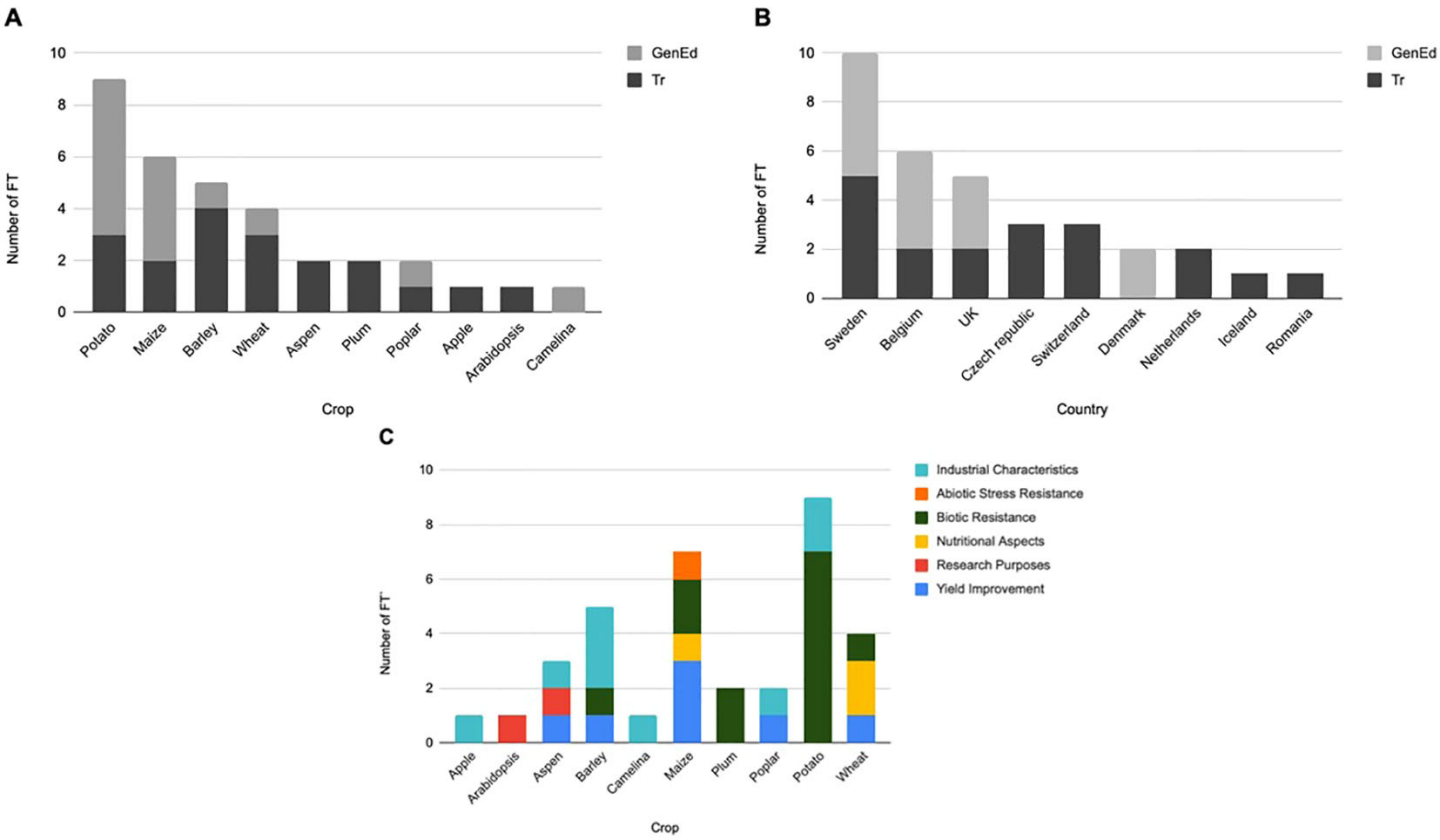
Number of FTs using GenEd and Tr crops in the EU, Iceland, Switzerland and UK (2022-2023). **(A)** Listed by crops; **(B)** Listed by countries; **(C)** Listed by traits.

Data were collected from 33 countries of geographical Europe (including non-EU countries) (**Figure 3**). Among the 11 countries performing FTs, only 9 countries provided data precise enough to be analyzed. In the non-EU countries Ukraine and Albania, FTs are forbidden (no details are available). **Figure 4A** shows that for most crops both GenEd and Tr are used. Potato is the most field-tested crop among the 10 crops listed and it is mostly for GenEd products. In Europe, Belgium, Sweden, and UK appear as the most innovative countries as revealed by both the total number of FTs and the testing of GenEd products (**Figure 4B**). One can note that Denmark is focusing its FTs on GenEd techniques. Among the 31 FTs, the transgenic (Tr) technique was still used in 58% of the cases (18 FTs). Traits modified are yield improvement (22.9% of total), research purposes (5.7%), nutritional aspects (8.6%), industrial characteristics (25.7%), biotic stress (34.3%) and abiotic stress resistance (2.9%) (**Figure 4C**).

#### 3.2.2 Regulatory status of FTs in Europe

FTs authorization procedures in the EU are defined in Part B (deliberate release of GMOs for any other purpose than for placing on the market) of Directive 2001/18/EC of the European Parliament and of the Council of 12 March 2001. The current political context is that some EU countries perform FTs with biotech crops, while in other countries such FTs are not permitted (see **Figure 3**). In addition, since the Court of Justice of the EU ruled on July 25th, 2018 that organisms obtained by modern forms of mutagenesis are not exempt from the EU GMO legislation (EU Directive 2001/18), GenEd crops, like transgenic ones are considered as GMOs.

##### 3.2.2.1 Countries where FTs are/were forbidden

In France, FTs are not prohibited by law. However, according to a report from the French Ministry of Agriculture, “No field experimentation of genetically modified plants is currently authorized in France. The last GM field trial in France was in 2013. No application for authorization has been filed since then”, hence the situation of Tr and GenEd FTs can be likened to a de facto ban. This is why French researchers and private companies have been in the recent past forced to relocate their trials in the UK or in Argentina, for example. The situation could change due to the start of a new national “priority research and equipment program” devoted to “advanced plant selection”, meaning “to select new species and new traits favorable to an agroecological transition and adaptation to climate change”. It should also be mentioned that the country prohibits by law cultivation of all “genetically modified” maize since 2014. This de facto includes edited maize varieties since the above-mentioned ruling of Court of Justice of the EU on July 25th, 2018. However, the French Minister of Agriculture Marc Fesneau declared in May 2023 to be in favor of the deregulation of GenEd plants and thus avoiding the long and costly authorization process for these plants currently classified as GMOs (as imposed by European Regulation 1829/2003 of 22 September 2003 and Commission Implementing Regulation No 503/2013 of 3 April 2013).

Germany, the country where the headquarters of major players in agricultural biotechnology such as Bayer CropScience or BASF are located, appears colored in red on the **Figure 3**. It is in a similar regulatory situation as France or Italy. Germany has “opted out” of the cultivation of the only transgenic event authorized in the EU (maize MON810). Nevertheless, FTs took place until 2017. The cessation of these trials has nothing to do with regulations and was more of a political choice of the new government at this time, which was strongly opposed to biotech crops. Even before the end of FTs in Germany, due to the rejection of GMs on the EU market, the large firms mentioned above moved their R&D centers outside the EU.

Biotech crops are not allowed to be cultivated in Italy as well, since it “opted out” (EC Directive 2015/412) in 2015. However, some research is conducted on tomato, olive and grapevine. The public opinion made it difficult to defend biotech plants for cultivation and for research purposes. This situation resulted in defunding of research and development of biotech crops. However, the regulation recently changed in favor of FT acceptance since in June 2023 Italian political groups voted unanimously to authorize field experimentation of products of new breeding technologies (NBTs) (ISAAA, 2023).

##### 3.2.2.2 European countries where FTs are authorized but no FTs were performed in 2022-2023

Spain is the biggest producer of biotech crops in the EU and defended a non-opt-out position in 2015. FTs are allowed, but notifications to the EC remain low as a reflection of a lack of interest to develop biotech crops adapted to the biotic and abiotic conditions of the country (Guerrero, 2023; OECD 2024). The same stands for Portugal where the cultivation of biotech plants is authorized for research purposes (Directive 2001/18/EC), but the reluctance of the farmers and the public for new biotech crops make it uninteresting to develop such plants.

##### 3.2.2.3 European countries where FTs were performed in 2022-2023

The United Kingdom (UK) is probably one of the most pro-innovation countries in Europe on this subject and especially on GenEd. Since Brexit, the UK Government has reduced the administrative burden on plant FTs. The Genetic Technology (Precision Breeding) Act 2023 (28 March 2023) has been passed by the British Parliament to reduce the regulatory burden on genome-edited plants, thus distinguishing them from conventional GMOs and facilitating research. As a consequence of this new policy, in April 2022, the Crop Science Centre, announced planting of a FT of Tr and GenEd barley with the aim of reducing dependency on synthetic fertilizers to promote improved soil health, and sustainable and equitable means of food production. Another country where FTs were performed was Switzerland where FTs are performed in dedicated and protected sites with fences (barley, maize and wheat resistant to biotic stress, see **Supplementary Table S5**).

The above-mentioned examples highlight that among opposing countries, there is not necessarily a regulation that prohibits FTs, sometimes the socio-political context pushes the governments to prevent the development of these tests even in the absence of a clear law prohibiting FTs. “The national media debate on biotech crops and plant experimentation has made it politically unpalatable to support biotech research and cultivation. Therefore, public and private research funding on biotech products has gradually been cut to zero and currently no biotech field trials are being conducted in Italy” (USDA, 2020).

#### 3.2.3 Status of FTs according to the proposed new regulation for GenEd products

The EU institutions are currently debating on the status of GenEd products (termed ‘new genetic techniques’, NGT), with a proposed different classification dependent on the nature of the modification. Basically, minor mutations that could have occurred ‘naturally’ would have a permissive NGT-1 status, while others would have the NGT-2 status (with still a heavy regulatory burden).

Whether the current FTs in the EU fall in one or the other category was determined (see **Supplementary Table S10**). Interestingly all collected FTs fall in the NGT-1 category. This trend holds true for the most recent FTs (see update in **Supplementary Table S10**). Two explanations can be proposed (which are not mutually exclusive): EU laboratories may anticipate that NGT-2 products may be difficult to bring to the European market and are avoiding developing such products; transgenesis may still be the preferred techniques for larger genome modifications such as DNA insertions. Belgium, Denmark and Sweden are the first EU countries where edited crops are tested in field trials. Belgium, Denmark and Sweden have always loosened the rules on biotech techniques (including gene editing) along with the UK.

### 3.3 Approvals for cultivation

To examine whether FTs bear new innovation promises with respect to recently authorized biotech events, we collected information on the latter crops and traits in order to compare this data with those of FTs over the same period. All events approved for cultivation between 2021 and 2023 are Tr events. No trait was obtained by GenEd, but this conclusion does not take USA into account, since in this country the events authorized for cultivation are not accessible in the USDA database. The EUGINIUS database includes biotechnology trials, but it is not complete for Europe (https://www.euginius.eu). No database is provided for the USA. The EUGINIUS database lists only a few trials conducted in the USA.

**Figure 5:**
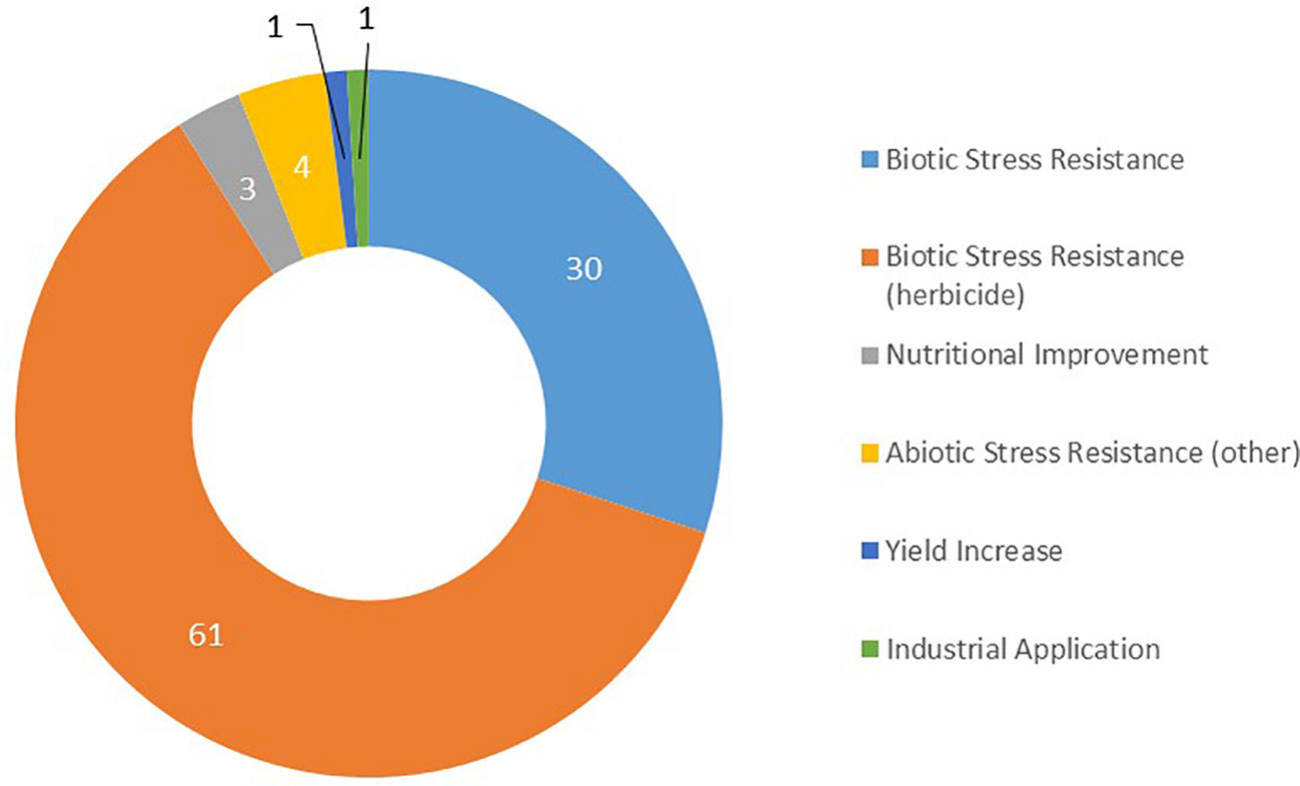
Number by category of traits of biotechnological crops approved for cultivation during years 2022-2023.

Since 2021 there were 198 authorized events that were related to 16 different plant species, while during the same period 36 crops were tested in FTs. Regarding traits, it can be noted that more than 90% of the events approved during the years 2022 and 2023 are related to the response to a biotic stress, and two thirds of these crops are tolerant to at least one herbicide (**Figure 5**). In **Figure 5**, all the events approved for cultivation are cited with percentages for abiotic stress resistance (4%), nutritional improvement (3%), yield increase (1%) and industrial application (1%). All data (countries and crops) are presented by trait with percentages in **Supplementary Tables S2**–**S8**. This data contrasts with the FT data shown in **Figure 2**.

To clarify the data on events authorized for cultivation, we have also subdivided them for one trait category per region. Data analysis for Africa shows that there are few countries in Africa, notably Ghana, Kenya, South Africa, releasing information on biotech crops cultivation, with South Africa emerging as the leader in the field of Tr crops (**Supplementary Tables S11**, **S12**). Insect resistant cowpea is now a new biotech crop in Ghana obtained by the public sector. Biotic stress resistance is developed by both the public and private sectors. However, in Africa, the lack of information and the absence of clear regulations make it quite difficult to analyze this information in depth.

Analysis of the available data for America excluding USA showed that a few biotech crops have been recently authorized for pests and diseases resistance in some American countries (cotton, maize, soybean, sugarcane - **Supplementary Table S13**). This category of traits is mostly developed by private companies. Canada is the most active country with 6 different herbicide-resistant crops approved for cultivation including herbicide tolerant sorghum, for adaptation to climate change (**Supplementary Table S14**). In Columbia stacked herbicide tolerant events (up to 3 herbicides and insect resistance event) are developed in cotton, maize and in soybean. Argentina, Brazil and Costa-Rica and Paraguay also authorized staked herbicide-tolerant events. Papaya was already approved in the USA; this is not recent. In the **Supplementary Table S13** recent crops approved for cultivation in Americas in 2022 and 2023 modified for biotic stress resistance by country are listed and no papaya was tested.

In Asia-Pacific most of authorized biotic stress resistance events relate to a few crops (cotton, maize, potato and soybean), while herbicide-tolerant canola, cotton, maize and soybean were authorized in most countries and wheat in Indonesia (**Supplementary Tables S15**, **S16**).

### 3.4 Bibliographic review and bibliometric analysis of FTs with GenEd plants

**Figure 6:**
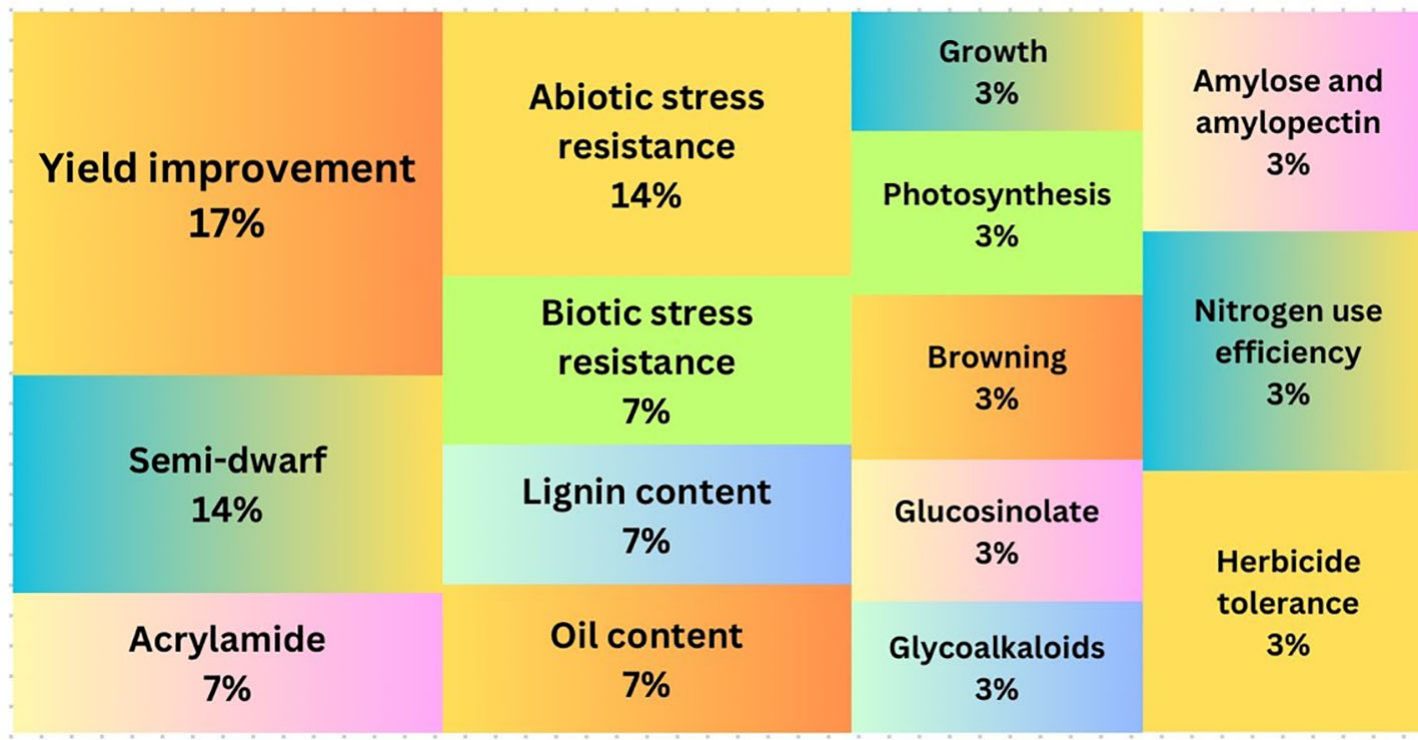
Keyword analysis results showing the most used traits.

A bibliographic search of academic articles mentioning FTs and GenEd was conducted for the 2018-2023 timeframe and 33 articles were found. The keyword analysis demonstrated that yield improvement and stress resistance were the most studied traits followed by other industrial properties (including semi-dwarf, acrylamide production and oil content, etc.). Lignin content, growth, photosynthesis, nitrogen use efficiency, browning, herbicide tolerance, glycoalkaloids, glucosinolate, amylose and amylopectin were other notable traits studied in FTsof biotech crops (**Figure 6**). When the content of each article was screened for method detection, CRISPR appeared in most of these 33 articles, while TALEN for example was never mentioned. Cas9 appeared in most cases when CRISPR was mentioned, and Cas12a only once. The term NGT (new genetic techniques) officially used by the European Commission occurred in 3 of these papers.

When collaborations between laboratories were analyzed, it appeared that most of them are within a single country (**Supplementary Figure S1**). Scientific interactions between laboratories from an African country and European countries appeared limited. No general trend has been identified suggesting that European laboratories have delocalized their FTs to permissive countries, when they were banned in their own country. This suggests that most European laboratories have simply not conducted FTs, or have not yet been able to publish their results.

## 4 Discussion

From the 141 countries for which data on FTs were searched, 41 were identified as performing FTs, involving 36 plant species. We were able to gather precise information on 30 of these countries. It seems that favorable regulation of biotech technologies in some countries outside the EU have boosted the development of biotech plants, which then could be tested in the fields without difficulties (Guo et al., 2023; Molitorisová et al., 2023; Zhong et al., 2024). However, in some cases, plants are deregulated under local regulations and therefore not specifically declared in public databases, so we could not compile them, a problem already encountered by other authors dealing with the subject (Metje-Sprink et al., 2020).

FTs with biotech crops are conducted by the public sector in 66% of the evaluated cases. However, it should be mentioned that the private sector is highly represented in application of GenEd for traits like herbicide tolerance and industrial applications, while the public sector is more involved in all other topics. In some MS in the EU, due to a certain reticence in view of vandalism and the regulations in EU, the development of new biotech crops is slowed down. In that context, some countries are carrying out confined laboratory research while no FTs are currently performed for political reasons.

In all countries surveyed, setting and presence of FTs with biotech crops was strongly affected by existing policies and regulations, as already observed and discussed by different authors (Metje-Sprink et al., 2020; Goralogia et al., 2021; Marone et al., 2023). The number of EU countries performing FTs was actually decreasing since 2015 (Ricroch, 2020). In our survey, we confirmed such a decrease of FTs in the EU. In Spain, the consents were given recently for two field trials for GE plants: B/ES/23/36 - a gene-edited tobacco line with high anatabine content and B/ES/21/28 - for increased salinity and drought tolerance in broccoli. In both cases, the mutant lines were generated using the CRISPR/Cas9 system. The tobacco edited line should be used as an anatabine biofactory, while the broccoli line should be more resistant to salinity and drought, without repercussions on other commercial qualities. Their potentials will be evaluated in field trials (OECD, 2024). The number of FTs and the diversity of crops among them have been decreasing since 2015. Potato and maize are mostly tested in field trials, but there have also been releases of wheat, barley, tobacco, poplar, oilseed rape and others (https://www.testbiotech.org/wp-content/uploads/2024/08/Field_trials_New_GE_EU_UK_background_17_08_2024.pdf). Since 2016, the total number of FT in EU and UK together has risen to about 50 with 13 plant species involved (https://webgate.ec.europa.eu/fip/GMO_Registers/GMO_Summary.php?NotificationNum=B/IT/24/04&Cat=gmp).

Worldwide, biotech events recently authorized for cultivation were mostly developed by private companies, and a few by the public sector in Brazil, China, Costa Rica, Ghana, and Kenya. This contrasts with the data compiled on FTs, which were mostly conducted by the public sector. Study of the approvals for cultivation highlighted the differences in crops and traits between the recently authorized events and those which may be authorized in the future following FTs over the period 2022-2023. The 198 events authorized for cultivation that we recorded since 2021 are related to 16 crops, mostly for herbicide tolerance (61%) and biotic stress resistance (30%). During the same period 36 crops were tested in FTs.

Our study showed that although a multitude of traits have been introduced into the crops using GenEd, and certain number of those crops tested in FTs, only few GenEd crops have reached the market so far, especially in the EU. This might be due to different regulations on GenEd crops in different countries, with some being more open to this new technology (Menz et al., 2020). A notable example of the effect of regulation on biotech crop cultivation is the new drought-resistant wheat (HB4), developed by Trigall Genetics, a Argentine Bioceres company’s joint venture, with the French company Florimond Desprez. Although developed by a European company, HB4 wheat is not commercialized in the Europe, but it is approved for cultivation in several countries outside EU. This adds the value to the argument, already raised by several other authors, that strict biotech policies and hurdles for FTs and cultivation of biotech crops will leave Europe lagging behind other countries, especially the USA and China, when it comes to market-oriented trait development using biotech techniques, making an even wider gap between research and application (Modrzejewski et al., 2019; Menz et al., 2020). However, a new regulatory policy in the EU could provide favorable conditions to the public and private sectors to innovate. While on 25 July 2018 the Court of Justice of the European Union ruled that some mutagenesis procedure (which includes GenEd) should be regulated like GM plants (case C-258/16), a more favorable, at least for some mutagenesis products (see above the distinction between NGT-1 and NGT-2), “proposal for a regulation of the European parliament and of the Council on plants obtained by certain new genomic techniques” has been published by the European Commission (EC) in July 2023 (Ricroch et al., 2024). An amended version of this proposal has been voted by the European parliament and is waiting for an agreement amongst MS.

In Japan, in 2020, the Animal Plant Health Inspection Service has determined that GenEd products are not regulated under 7 CFR part 340. In Senegal a new law in 2022 authorized biotech research.

The diversity of crops and traits tested in the field is higher than in approved events during the same period. In addition, while the latter used mostly Tr, GenEd is used in 12% of FTs (a number which is underestimated since in the USA data on GenEd are not all publicly released). It can be concluded from the present compilation that biotech of plants holds great promises, despite the fact that not all field-tested products will make it to the market.

The global “regulatory mixture” hampers the global release, but also import and export of GenEd plants, so the first GenEd crops released so far were marketed in countries with a GenEd friendly policy. Moreover, we have identified that most countries, which are active in developing market-oriented traits have such a friendly policy (e.g., United States and Japan), with China and the United States leading this field. Europe which has a strict policy toward GenEd is among the leaders in GenEd research (Modrzejewski et al., 2019) but is lagging in the application of this technology. However, a changing regulatory landscape, along with emerging studies on novel GenEd tools are focused on transgene-free editing, which are deemed to be more ‘regulatory-friendly’ and may attract improved public approval, should result in an increasing number of biotech plants tested in the field, especially in the EU (Metje-Sprink et al., 2020; Miladinović et al., 2021; Hamdan et al., 2022) and possibilities to reach the market. It should be kept in mind that even if some GenEd crops are deregulated, it will still be crucial to be able to perform FTs on these crops, firstly to verify that a given trait is satisfactorily expressed in real conditions (not only in a confined environment) and secondly to comply with intellectual property rules (such as to obtain a COV for example, as mentioned in the Introduction).

## Data Availability

The original contributions presented in the study are included in the article/**Supplementary Material**. Further inquiries can be directed to the corresponding author.

## Supplementary material

Supplementary Methods S1 Survey and statistics associated: 57 Countries answered the survey, the data was sufficient to be processed for 55 of them.

Supplementary Table S1 Data on biotech laboratory research carried out by country (141 countries); NA – No information found

Supplementary Table S2 Status of FTs by country in 89 countries. Sources: our survey and OECD, PC, EC, FAO, GAIN and GVDB databases (see Methods); *In 2022 and 2023

Supplementary Table S3 Crops tested in FTs in 2022 and 2023 for herbicide tolerance by country. Pvt – Private; Pub – Public; * - developed by genome editing (GenEd); NA – data not available

Supplementary Table S4 Crops tested in FTs in 2022 and 2023 for abiotic stress by country. Pvt – Private; Pub – Public; * - developed by genome editing (GenEd); NA – data not available

Supplementary Table S5 Crops tested in FTs in 2022 and 2023 for biotic stress, by country. Pvt – Private; Pub – Public; * - developed by genome editing (GenEd); NA – data not available.

Supplementary Table S6 Crops tested in FTs in 2022 and 2023 for quality improvement by country. Pvt – Private; Pub – Public; * - developed by genome editing (GenEd); NA – data not available.

Supplementary Table S7 Crops tested in FTs between 2022 and 2023 for industrial application by country. Pvt – Private; Pub – Public; * - developed by genome editing (GenEd); NA – data not available.

Supplementary Table S8 Crops tested in FTs between 2022 and 2023 for yield improvement by country. Pvt – Private; Pub – Public; * - developed by genome editing (GenEd).

Supplementary Table S9 Crops tested in FTs in 2022 and 2023 for research purposes by country. Pvt – Private; Pub – Public.

Supplementary Table S10 Crops tested in field trials in 2022 and 2023 modified by genome editing listed by country. Pvt – Private; Pub – Public.

Supplementary Table S11 Events approved for cultivation in Africa in 2022 and 2023 for biotic stress resistance by country. Pvt – Private; Pub – Public; NA – data on trait not available; IR – Insect resistance.

Supplementary Table S12 Crops approved for cultivation in Africa in 2022 and 2023 modified for traits regarding herbicide tolerance (HT) by country. Pvt – Private; Pub – Public; NA – data on trait not available; GLU – Glufosinate-ammonium resistance.

Supplementary Table S13 Crops approved for cultivation in Americas in 2022 and 2023 modified for biotic stress resistance by country. Pvt – Private; Pub – Public; NA – data on trait not available; IR – Insect resistance; NR – Nematode resistance.

Supplementary Table S14 Events approved for cultivation in America between 2022 and 2023 modified for herbicide tolerance by country. Pvt – Private; Pub – Public; * - events stacked with IR events; NA – data on trait not available; GLU – Glufosinate resistance; GLY – Glyphosate resistance; IFT – Isoxaflutol resistance; DC – Dicamba resistance; HPPD - Hydroxyphenylpyruvate dioxygenase inhibiting herbicide family resistance; ACC - ACCase inhibitor resistance; IR – Insect resistance; NR – Nematode resistance.

Supplementary Table S15 Events approved for cultivation in Asia-Pacific between 2022 and 2023 for biotic stress resistance by country. Pvt – Private; Pub – Public; IR – Insect resistance; NR – Nematode resistance; BR – Bacterial resistance.

Supplementary Table S16 Events approved for cultivation in Asia-Pacific between 2022 and 2023 modified for herbicide tolerance by country. Pvt – Private; Pub – Public; * - events stacked with IR events; NA – data on trait not available; GLU – Glufosinate resistance; GLY – Glyphosate resistance; IFT – Isoxaflutol resistance; DC – Dicamba resistance; HPPD - Hydroxyphenylpyruvate dioxygenase inhibiting herbicide family resistance; ACC - ACCase inhibitor resistance.

Supplementary Figure S1 Collaboration network analysis among the most active institutions on the topic. Collaboration threshold set for < 1 paper.
