# Corrigendum: Worldwide study on field trials of biotechnological crops: new promises but old policy hurdles

**DOI:** 10.3389/fpls.2024.1531017

**Published:** 2024-12-06

**Authors:** Agnès Ricroch, Louie-David Desachy, Mateo Penfornis, Melekşen Akin, Ankica Kondić-Špika, Marcel Kuntz, Dragana Miladinović

**Affiliations:** ^1^ Laboratoire Institut Droit, Espaces et Technologies (IDEST), Faculté Jean Monnet, Université Paris-Saclay, Sceaux, France; ^2^ AgroParisTech, Université Paris-Saclay, Palaiseau, France; ^3^ Department of Horticulture, Agricultural Faculty, Iğdır University, Iğdır, Türkiye; ^4^ Laboratory for Biotechnology, Institute of Field and Vegetable Crops, National Institute of Republic of Serbia, Novi Sad, Serbia; ^5^ Laboratoire de Physiologie Cellulaire et Végétale, Université Grenoble Alpes, Centre national de la recherche scientifique (CNRS), Commissariat à l’énergie atomique et aux énergies alternatives (CEA), Institut national de recherche pour l’agriculture, l’alimentation et l’environnement (INRAE), Grenoble, France

**Keywords:** transgenesis, CRISPR, genome editing, plant breeding, field trials, biotechnology regulatory policy

In the published article, there was an error in **Figure 4** as published. The sections A, B and C overlap and legends cannot be read. The corrected **Figure 4** appears below.

**Figure 4:**
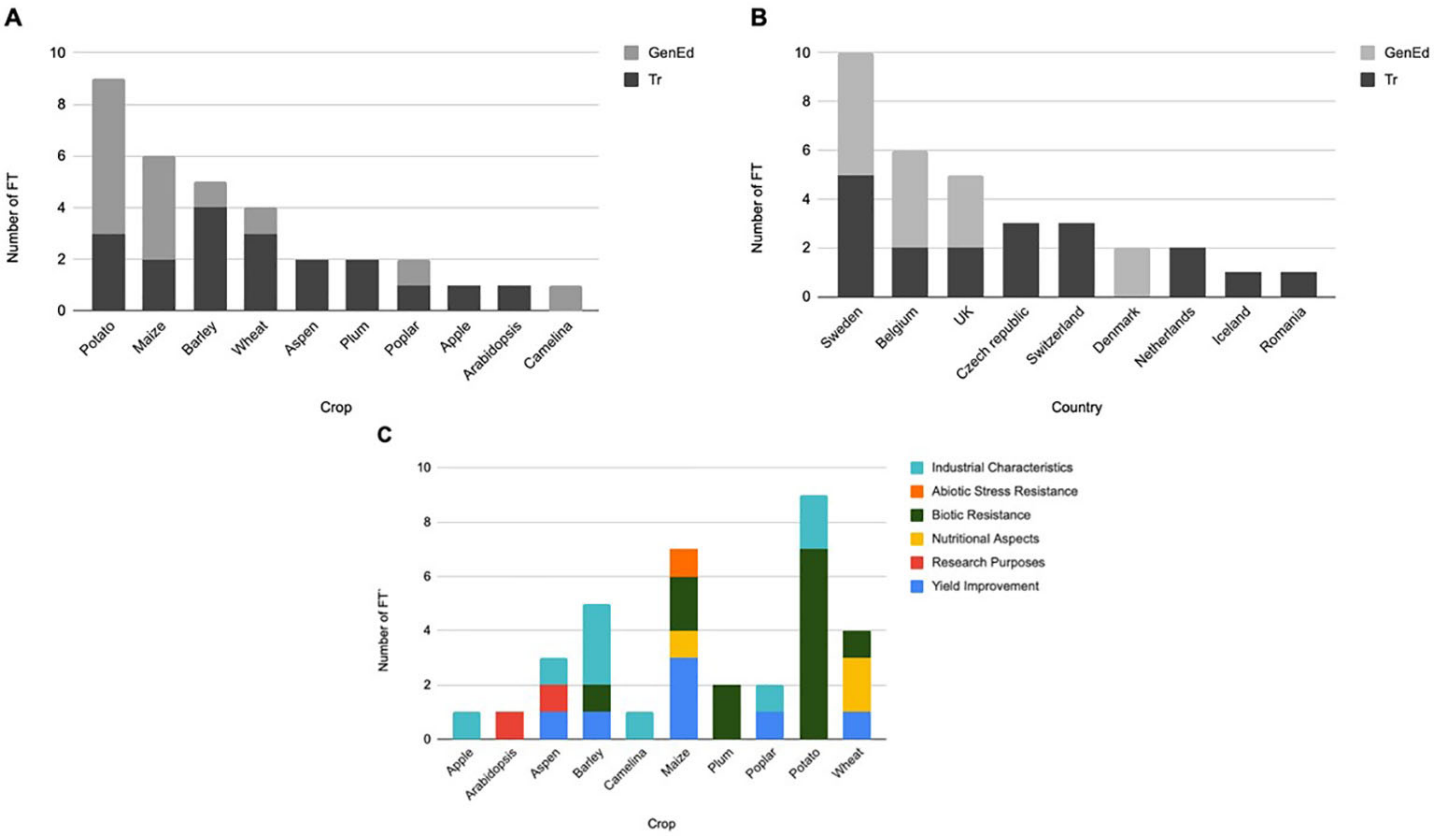
Number of FTs using GenEd and Tr crops in the EU, Iceland, Switzerland and UK (2022-2023). **(A)** Listed by crops; **(B)** Listed by countries; **(C)** Listed by traits.

In the published article, there was an error in the **Supplementary Material**. **Supplementary Tables S3**, **S4**, **S8**, **S12** and **S15** were cut by page separation.

The authors apologize for these errors and state that this does not change the scientific conclusions of the article in any way. The original article has been updated.

