# Supplementary material for: Worldwide study on field trials of biotechnological crops: new promises but old policy hurdles
Source: Front Plant Sci. 2024 Nov 4;15:1452767. doi: 10.3389/fpls.2024.1452767 (PMC11570883; doi:10.3389/fpls.2024.1452767)
Supplement: Supplementary Methods S1 — Survey and statistics associated: 57 Countries answered the survey, the data was sufficient to be processed for 55 of them. [file DataSheet1.pdf]

Supplementary Material

Worldwide Study on Field Trials of Biotechnological Crops: New Promises but Old Policy Hurdles

Agnès Ricroch[1,2], Louie-David Desachy[2], Mateo Penfornis[2], Melekşen Akin[3], Ankica Kondić-Špika[4], Marcel Kuntz[5], Dragana Miladinović[4*]

*** Correspondence:** Dragana Miladinović: dragana.miladinovic@ifvcns.ns.ac.rs

Methods S1 Survey and statistics associated: 57 Countries answered the survey, the data was sufficient to be processed for 55 of them.

Your country:

- Q1

Is there GMO regulation(s) in your country?

- Q2

Are GenEd plants also covered by the GMO legislation in your country?

  - Other, please explain below:

- Q3

Are field trials regulated by this regulation(s)?

- Q4

Are field trials allowed by this regulation(s)?

- Q5

Are there currently field trials of Tr/GenEd plants in your country?

- Q6

Is there a national website collecting these trials?

  - Please provide the link, if it exists.

- Q7

If there are no field trials in your country, what are the reasons for this?

  - Other, please explain below:

- Q8

In your opinion, what impact could have the lack of field trials on agri-food sector and relevant R&I activities in your country?

    - Negative
    - Lagging behind other countries such as US and China
    - Narrowed possibility to achieve Green Deal goals
    - Narrowed possibility to have local products/row materials available
    - Other, please explain below
- Q9

Would you personally need such trials for you own work?

Multiple Choice

    - Not personally, but other colleagues/groups do
    - Please name colleague/group needing field trials:
- Q10

Why do you value such trials?

Multiple Choice

    - Valuable for proof of concept
    - For R&D activities
    - For pre-marketing activities
    - For evaluation in local environmental conditions
    - Other, please explain below:
- Q11

If trials are not possible in your country, do you consider performing these trials in another country?

Multiple Choice

1 Supplementary Figures and Tables

1.1 Supplementary Tables

Table S1 Data on biotech laboratory research carried out by country (141 countries); NA – No information found

**Country**	**Laboratory research**	**Country**	**Laboratory research**
Afghanistan	NA	Liberia	NA
Albania	No	Lithuania	NA

Algeria	Yes	Luxembourg	Yes
Angola	NA	Madagascar	NA
Argentina	No	Malawi	NA
Armenia	NA	Malaysia	NA
Australia	Yes	Maldives	NA
Austria	NA	Mali	NA
Azerbaijan	NA	Malta	NA
Bahrain	NA	Mauritania	NA
Bangladesh	NA	Mauritius	Yes
Belgium	Yes	Mexico	NA
Benin	NA	Moldova	NA
Bhutan	No	Mongolia	NA
Bosnia and Herzegovina	NA	Montenegro	NA
Botswana	NA	Morocco	NA
Brazil	Yes	Mozambique	Yes
Brunei	Yes	Myanmar	NA
Bulgaria	Yes	Namibia	NA
Burkina Faso	NA	Nepal	NA
Burundi	NA	Netherlands	Yes
Cabo Verde	Yes	New Zealand	Yes
Cambodia	NA	Nicaragua	NA
Cameroon	Yes	Niger	NA
Canada	Yes	Nigeria	Yes

Central African Republic	NA	Norway	Yes
Chad	NA	Pakistan	Yes
Chile	Yes	Panama	NA
China	Yes	Peru	NA
Colombia	Yes	Philippines	Yes
Comoros	NA	Poland	NA
Congo	NA	Portugal	Yes
Costa Rica	Yes	Qatar	NA
Côte d'Ivoire	NA	RDC	NA
Croatia	No	Romania	Yes
Cyprus	No	Russia	Yes
Czech Republic	Yes	Rwanda	NA
Denmark	NA	Saudi Arabia	NA
Djibouti	NA	Senegal	Yes
Dominican Republic	NA	Serbia	Yes
Ecuador	NA	Sierra Leone	NA
Egypt	Yes	Singapore	Yes
Eritrea	NA	Slovakia	NA
Estonia	NA	Slovenia	NA
Eswatini	NA	Somalia	NA
Ethiopia	NA	South Africa	Yes
Finland	Yes	South Korea	Yes
France	Yes	South Sudan	NA
Gabon	NA	Spain	Yes

Gambia	NA	Sri Lanka	Yes
Georgia	NA	Surinam	NA
Germany	Yes	Sweden	Yes
Ghana	Yes	Switzerland	Yes
Greece	NA	Syria	NA
Guatemala	NA	Taiwan	Yes
Guinea Bissau	NA	Tanzania	Yes
Honduras	Yes	Thailand	NA
Hungary	Yes	Timor-Leste	NA
Iceland	Yes	Togo	NA
India	Yes	Tunisia	Yes
Indonesia	Yes	Turkey	Yes
Ireland	NA	Uganda	Yes
Israel	Yes	Ukraine	NA
Italy	No	United Arab Emirates	Yes
Japan	Yes	United Kingdom	Yes
Jordan	NA	Uruguay	Yes
Kazakhstan	Yes	Uzbekistan	NA
Kenya	Yes	Vietnam	NA
Kyrgyzstan	No	Zambia	NA
Latvia	NA	Zimbabwe	Yes
Lesotho	NA	**Total**	**59**

Table S2 Status of FTs by country in 89 countries. Sources: our survey and OECD, PC, EC, FAO, GAIN and GVDB databases (see Methods); *In 2022 and 2023

	**Are FTs allowed in your country?**			**Are there FTs currently in your country?***	
**Country**	**Allowed**	**Approval needed**	**Not Allowed**	**Yes**	**No**
Albania			+		+
Algeria			+		+
Argentina		+		+	
Armenia			+		+
Australia		+		+	
Austria		+			+
Azerbaijan		+			+
Bangladesh		+		+	
Belgium		+		+	
Bosnia and Herzegovina			+		+
Brazil		+		+	
Bulgaria		+			+
Burkina Faso		+			+
Canada	+			+	
Chile		+		+	
China	+			+	
Colombia		+		+	

Costa Rica		+		+	
Croatia		+			+
Cyprus			+		+
Czech Republic		+		+	
Denmark		+		+	
Dominican Republic			+		+
Ecuador			+		+
Egypt	+			+	
Estonia			+		+
Ethiopia	+			+	
Finland		+			+
France			+		+
Georgia		+			+
Germany		+			+
Greece			+		+
Guatemala		+		+	
Honduras		+		+	
Hungary		+			+
Iceland		+			+

India		+			+
Indonesia		+		+	
Ireland			+		+
Israel		+		+	
Italy		+			+
Japan		+		+	
Jordan			+		+
Kazakhstan		+		+	
Kenya		+		+	
Latvia		+			+
Lithuania		+			+
Malaysia		+		+	
Mexico			+		+
Moldova		+		+	
Morocco			+		+
Mozambique		+		+	
Netherlands		+		+	
New-Zealand		+		+	
Nicaragua			+		+

Nigeria		+		+	
Norway		+			+
Pakistan		+			+
Peru		+			+
Philippines		+		+	
Poland		+			+
Portugal			+		+
Qatar			+		+
Romania		+			+
Russia	+			+	
Saudi Arabia		+			+
Senegal		+			+
Serbia		+			+
Slovakia		+			+
Slovenia		+			+
South Africa		+		+	
South Korea		+		+	
Spain		+		+	
Sri Lanka		+		+	
Surinam			+		+

Sweden		+		+	
Switzerland		+		+	
Taiwan		+		+	
Tanzania			+		+
Tunisia		+		+	
Turkey			+		+
UAE		+		+	
Uganda	+				+
UK		+		+	
Ukraine			+		+
Uruguay		+		+	
USA		+		+	
Venezuela			+		+
Zimbabwe			+		+
**Total**	**6**	**60**	**23**	**41**	**48**

Table S3 Crops tested in FTs in 2022 and 2023 for herbicide tolerance by country. Pvt – Private; Pub – Public; * - developed by genome editing (GenEd); NA – data not available

	**Camelina**	**Canola**	**Cotton**	**Grass**	**Maize**	**Mustard**	**Pine**	**Poplar**	**Potato**	**Soybean**	**Wheat**
**Australia**		Pvt				Pvt					

**Canada**	Pvt	Pvt			Pvt					Pvt	Pvt
**Colombia**		Pvt			Pvt						
**India**			Pub		Pub						
**Indonesia**									Pub*		
**New-Zealand**							Pub				
**Pakistan**			Pvt								
**South Korea**		NA		Pub							
**USA**					Both*			Pub		Both*	

Table S4 Crops tested in FTs in 2022 and 2023 for abiotic stress by country. Pvt – Private; Pub – Public; * - developed by genome editing (GenEd); NA – data not available

	**Barley**	**Canola**	**Chickpea**	**Cotton**	**Grass**	**Maize**	**Rice**	**Soybean**	**Sugarcane**	**Wheat**
**Australia**	Pub		Pub							Pub
**Belgium**						Pub*				
**Canada**		Pub						Pvt		
**Indonesia**							Pub*			
**Pakistan**				Pub						
**South Korea**							NA			
**UAE**					Pub					

**USA**	Pub

Table S5 Crops tested in FTs in 2022 and 2023 for biotic stress, by country. Pvt – Private; Pub – Public; * - developed by genome editing (GenEd); NA – data not available.

	**Barley**	**Beans**	**Canola**	**Chili**	**Citrus**	**Cotton**	**Cowpea**	**Maize**	**Pepper**	**Poplar**	**Plum**	**Potato**	**Grass**	**Rice**	**Soybean**	**Vine**	**Walnut**	**Wheat**
**Australia**																		Pub
**Canada**			Pvt					Pvt							Pvt			Pvt
**Czech Republic**											Pub							
**Denmark**												Pvt *						
**India**						Pub		Pub										
**Indonesia**				Pub *	Pub *									Pub *				
**Kazakhstan**												Pub						
**Kenya**												NA						
**Netherlands**												Pub						
**Nigeria**							Pub					Pub						
**Pakistan**						Pub												
**Romania**											Pub							
**South Korea**		NA							NA					NA				

**Sweden**												Both*						
**Switzerland**	Pub							Pub										Pub
**United Kingdom**												Pub						
**Uruguay**													Pub					
**USA**	Pub				Both*			Pvt		Pub						Pub	Pub	Pub

Table S6 Crops tested in FTs in 2022 and 2023 for quality improvement by country. Pvt – Private; Pub – Public; * - developed by genome editing (GenEd); NA – data not available.

	**Apple**	**Banana**	**Beans**	**Camelina**	**Canola**	**Citrus**	**Clary S[illegible]**	**Maize**	**Mustard**	**Pineapple**	**Potato**	**Rice**	**Sorghum**	**Soybean**	**Walnut**	**Wheat**
**Australia**					Pvt				Pvt							Pub
**Belgium**								Pub*								
**Canada**					Pvt						Pub			Pvt		
**Costa Rica**										NA						
**Honduras**		Pvt														
**Philippines**												Pub				
**Sweden**																Pub
**South Korea**	NA		NA									NA				
**United Kingdom**																Pub

**USA**	Pub	Pub*	Pvt	Pvt	Pub

Table S7 Crops tested in FTs between 2022 and 2023 for industrial application by country. Pvt – Private; Pub – Public; * - developed by genome editing (GenEd); NA – data not available.

	**Apple**	**Aspen**	**Banana**	**Barley**	**Cabbage**	**Camelina**	**Canola**	**Grass**	**Maize**	**Poplar**	**Poppy**	**Potato**	**Rice**	**Sorghum**	**Sugarcane**
**Belgium**										Pub					
**Canada**				Pvt		Pvt	Pvt				Pvt				
**Czech Republic**				Pvt											
**Honduras**			Pvt												
**Iceland**				Pvt											
**Netherlands**	Pub														
**Sweden**		Pub										Pvt*			
**South Korea**					NA								NA		
**United Kingdom**															
**USA**								Pvt	Pvt				Pvt	Pub	Pub

Table S8 Crops tested in FTs between 2022 and 2023 for yield improvement by country. Pvt – Private; Pub – Public; * - developed by genome editing (GenEd).

	Aspen	Barley	Camelina	Cassava	Grass	Maize	Pine	Poplar	Rice	Soybean	Wheat
**Australia**		Pub									Pub
**Belgium**						Both*					
**Canada**			Pvt							Pvt	
**Indonesia**									Pub*		
**Japan**									Pub		
**New Zealand**							Pub				
**Nigeria**				Pub							
**Sweden**	Pub							Both*			
**United Kingdom**		Pub*	Pub*								Pub*
**Uruguay**					Pub						
**USA**					Both	Pvt		Pub*			

Table S9 Crops tested in FTs in 2022 and 2023 for research purposes by country. Pvt – Private; Pub – Public.

	Arabidopsis	Aspen	Grass	Pine Trees	Poplar	Sorghum	White Clover
**Australia**						Pub	Pvt

**Canada**						Pub		
**New-Zealand**					Pub			
**Sweden**	Pub	Pub						
**USA**				Pub				

Supplementary table 10. Crops tested in field trials in 2022 and 2023 modified by genome editing listed by country.

Notification Number for field trials in the EU, as they appear in

https://webgate.ec.europa.eu/fip/GMO_Registers/GMO_Part_B_Plants.php

are given between brackets.

	**Barley**	**Camelina**	**Citrus**	**Chili**	**Maize**	**Poplar**	**Potato**	**Rice**	**Soybean**	**Wheat**
**Belgium**					Pub/NGT-1 (B/BE/22/V1)<br>Pub/NGT-1 (B/BE/22/V2)<br>Pub:NGT-1 (B/BE/22/V3)<br>Pvt/NGT-1 (B/BE/23/V1)					
**Denmark**							Pvt/NGT-1 (B/DK/55/Waxy)<br>Pvt/NGT-1 (B/D			

							K/54/StDMR6-1)			
**Indonesia**			Pub	Pub			Pub	Pub		
**Sweden**						Pvt/NGT-1 (B/SE/21/22027)	Pub/NGT-1 (B/SE/22/23780)<br>Pub/NGT-1 (B/SE/23/3093)			
**United Kingdom**	Pub	Pub								Pub
**USA**			Pub		Pub	Pub			Pub	

Pvt – Private; Pub – Public; For FTs in the EU, the regulatory status (NGT-1; no NGT-2 identified) is given.

Recent update of Table 10 for the purpose of determining the NGT-1 or NGT-2 status of GenEd crops tested in Europeans FTs after May 2023 (listed by country).

	**Tobacco**	**Camelina**	**Citrus**	**Chili**	**Maize**	**Poplar**	**Potato**	**Rice**	**Soybean**	**Wheat**
**Belgium**					NGT-1 (B/Be/23/V4)<br>NGT-1 (B/BE/24/V3)					
**Denmark**							NGT-1 (B/DK/24/23110)			
**Spain**	NGT-1 (B/ES/									

	23/36)					
**Sweden**				NGT-1 (B/SE/24/5457)*<br>NGT-1 (B/SE/23/21689)**		
**Italy**					NGT-1 (B/IT/24/01)	

*13 lines in this FT: 9 are NGT-1, 4 are transgenics

*16 constructs in this FT, some NGT-1, some transgenics

Table S11 Events approved for cultivation in Africa in 2022 and 2023 for biotic stress resistance by country. Pvt – Private; Pub – Public; NA – data on trait not available; IR – Insect resistance.

	**Cotton**	**Cowpea**	**Maize**	**Soybean**
**Ghana**		IR (Pub)		
**Kenya**	NA (Pub)		NA (Pub)	
**South Africa**			NA (Pvt)	NA (Pvt)

Table S12 Crops approved for cultivation in Africa in 2022 and 2023 modified for traits regarding herbicide tolerance (HT) by country. Pvt – Private; Pub – Public; NA – data on trait not available; GLU – Glufosinate-ammonium resistance.

	**Cotton**	**Maize**	**Soybean**	**Wheat**

**Ghana**		NA (Pvt)	NA (Pvt)	
**Nigeria**				GLU (Pvt)
**South Africa**	NA (Pvt)	NA (Pvt)	NA (Pvt)	

Table S13 Crops approved for cultivation in Americas in 2022 and 2023 modified for biotic stress resistance by country. Pvt – Private; Pub – Public; NA – data on trait not available; IR – Insect resistance; NR – Nematode resistance.

	**Cotton**	**Maize**	**Soybean**	**Sugarcane**
**Argentina**		NA (Pvt)	NA (Pvt)	
**Brazil**	NA (Pvt)	NA (Pvt)	NA (Pvt)	NA (Pub)
**Canada**		IR (Pvt)	NR (Pvt)	
**Colombia**	NA (Pvt)	NA (Pvt)	NA (Pvt)	
**Costa Rica**	NA (Pub)			
**Paraguay**		NA (Pvt)	NA (Pvt)	

Table S14 Events approved for cultivation in America between 2022 and 2023 modified for herbicide tolerance by country. Pvt – Private; Pub – Public; * - events stacked with IR events; NA – data on trait not available; GLU – Glufosinate resistance; GLY – Glyphosate resistance; IFT – Isoxaflutol resistance; DC – Dicamba resistance; HPPD - Hydroxyphenylpyruvate dioxygenase inhibiting herbicide family resistance; ACC - ACCase inhibitor resistance; IR – Insect resistance; NR – Nematode resistance.

	Alfalfa	Camelina	Canola	Cotton	Eucalyptus	Maize	Rice	Sorghum	Soybean	Wheat
**Argentina**	GLY (Pvt)					DC+GLU+GL, GLU (Pvt)				NA (Pvt)
**Brazil**				NA (Pvt)	GLY (Pvt)	GLU+GLY (+IR*) (Pvt)			HPPD (+NR*) (Pvt)	NA (Pvt)
**Canada**		NA (Pvt)	NA (Pvt)			GLU (+IR*) (Pvt)	NA (Pvt)	NA (Pvt)	HPPD (+IR*) (Pvt)	
**Colombia**				GLY+(IR*), HPPD+(IR*), DC +GLY (Pvt)		GLY+GLU (+IR*), GLY (+IR*), GLU, DC+GLU+GLY (+IR*), 2,4D (+IR*), GLU (+IR*) (Pvt)			HPPD (+NR*), GLY (Pvt)	
**Costa Rica**				DC+GLU+ GLY+(IR*) (Pub)						
**Paraguay**						GLU+ GLY+(IR*) (Pvt)			HPPD (Pvt)	

Table S15 Events approved for cultivation in Asia-Pacific between 2022 and 2023 for biotic stress resistance by country. Pvt – Private; Pub – Public; IR – Insect resistance; NR – Nematode resistance; BR – Bacterial resistance.

	Cotton	Maize	Potato	Soybean

**China**	IR<br>(Both)	IR<br>(Both)		IR<br>(Pub)
**Indonesia**	IR<br>(Pvt)	IR<br>(Pvt)		IR, NR<br>(Pvt)
**Japan**		IR<br>(Pvt)	BR<br>(Pvt)	NR<br>(Pvt)
**South Korea**	IR (Pvt)	IR (Pvt)		

Table S16 Events approved for cultivation in Asia-Pacific between 2022 and 2023 modified for herbicide tolerance by country. Pvt – Private; Pub – Public; * - events stacked with IR events; NA – data on trait not available; GLU – Glufosinate resistance; GLY – Glyphosate resistance; IFT – Isoxaflutol resistance; DC – Dicamba resistance; HPPD - Hydroxyphenylpyruvate dioxygenase inhibiting herbicide family resistance; ACC - ACCase inhibitor resistance.

	**Canola**	**Cotton**	**Maize**	**Soybean**	**Wheat**
**Australia**	NA<br>(Pvt)				
**China**			ACC (+IR*)<br>GLY (+IR*) (Both)	NA<br>(Pub)	
**Indonesia**	IFT,<br>GLU<br>(Pvt)	IFT, GLY<br>IFT+GLY<br>(Pvt)	GLU<br>(Pvt)	GLU<br>(Pvt)	GLU<br>(Pvt)
**Japan**	DC<br>(Pvt)		GLU,<br>DC+GLY,<br>(Pvt)	HPPD<br>(Pvt)	
**South Korea**		GLY+GLU+DC (+IR*), ACC (+IR+NR*),<br>GLU+2,4-D<br>(+IR*),	GLY+GLU+DC;<br>(Pvt)		

GLU+HPPD (+IR*),
GLU (IR*),
(Pvt)

1.2 Supplementary Figures

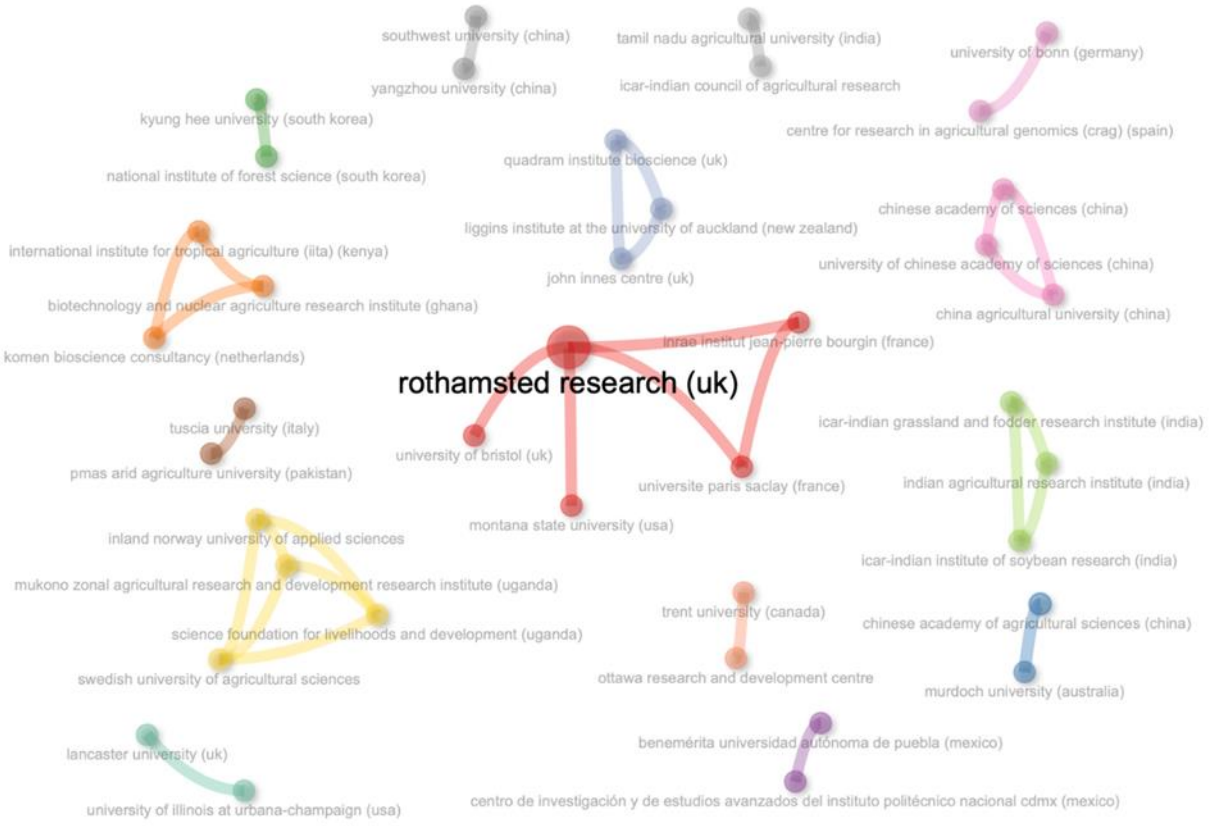

Supplementary Figure 1. Collaboration network analysis among the most active institutions on the topic. Collaboration threshold set for < 1 paper.
